# Transforming pediatric intensive care unit outcomes in pediatric hematology-oncology and hematopoietic stem cell transplantation through golden hour care in a low- and middle-income country: a 10-year analysis

**DOI:** 10.62675/2965-2774.20260291

**Published:** 2026-08-07

**Authors:** Indira Jayakumar, Milind Ballal, Nalla Anuraag Reddy, Rachit Mehta, Parvathy Menon, Ramya Uppuluri, Revathi Raj, Sandhiya Harikrishnan, Jennifer McArthur

**Affiliations:** 1 Pediatric Intensive Care Unit Apollo Cancer Centre Chennai India Pediatric Intensive Care Unit, Apollo Cancer Centre - Chennai, India.; 2 Apollo Cancer Centre Department of Paediatric Haematology, Oncology, Blood & Marrow Transplant Chennai India Department of Paediatric Haematology, Oncology, Blood & Marrow Transplant, Apollo Cancer Centre - Chennai, India.; 3 Apollo Cancer Centre Clinical Research Chennai India Clinical Research, Apollo Cancer Centre - Chennai, India.; 4 University of Tennessee Health Sciences Centre St Judes Children's Research Hospital Department of Pediatric Critical Care and Pulmonary Medicine Tennessee United States Department of Pediatric Critical Care and Pulmonary Medicine, St Judes Children's Research Hospital, University of Tennessee Health Sciences Centre - Tennessee, United States.

**Keywords:** Neonatal golden hour, Critical care, Intensive care units, pediatrics, Hematopoietic stem cell transplantation, Quality improvement, Developing countries

## Abstract

**Objective:**

To evaluate the impact of a care improvement bundle focused on early intervention, communication, and multidisciplinary collaboration by comparing outcomes across two 5-year periods before and after its implementation.

**Methods:**

A single-center, retrospective study was conducted in the Onco-Critical Care pediatric intensive care unit of a tertiary cancer referral hospital in South India, comparing outcomes from two eras: the pre-bundle cohort (2014 - 2018) and the post-bundle cohort (2019 - 2023). The study included children aged 1 month to 18 years, excluding those with postoperative, palliative, and end-of-life conditions. The care improvement bundle, introduced in 2019, aimed to enhance communication between oncology/transplant teams and the pediatric intensive care unit, enabling timely recognition and intervention ("golden hour care") for at-risk patients on the wards. The pre-bundle cohort had 4,000 hospital admissions and 533 pediatric intensive care unit admissions; the post-bundle cohort had 5,007 hospital admissions and 760 pediatric intensive care unit admissions.

**Results:**

From 2014 - 2018, 516 hematopoietic stem cell transplantations and 336 new oncology cases were managed; from 2019 - 2023, these increased to 579 hematopoietic stem cell transplantations and 784 new oncology patients. Despite increased pediatric intensive care unit admissions and a higher proportion of transplanted infants in the later cohort, pediatric intensive care unit mortality declined significantly from 21.8% to 11.4% (p < 0.0001). The use of costly interventions decreased, with invasive ventilation dropping from 30.9% to 18.0% (p < 0.0001) and renal replacement therapy from 14.8% to 4.3% (p = 0.0001), indicating both improved outcomes and reduced resource utilization.

**Conclusion:**

The implementation of a care improvement bundle to foster interdisciplinary collaborative communication and earlier recognition of critical illness enabled golden hour care on the floors, resulting in significant improvements in outcomes for pediatric oncology and hematopoietic stem cell transplantation patients. This care improvement bundle is feasible, easily scalable, and can be systemically incorporated across the centers.

## INTRODUCTION

Over the past few decades, advancements in cancer treatments have improved our understanding of pediatric malignancies and led to better outcomes for children. With its expanding indications, Hematopoietic stem cell transplantation (HSCT) is now an established curative option for both malignant and non-malignant conditions, including hemoglobinopathies, haematologic and solid tumors, immunodeficiencies, and genetic and metabolic disorders.

Children undergoing HSCT or receiving intensive treatment for hematological-oncological conditions often face aggressive therapies that increase their risk of severe complications. It is estimated that about 40% of these patients will need intensive care at some point during their treatment. The overall pooled mortality rate for this group remains high, at 27.8% (95% confidence interval [95%CI] 23.7 - 31.9%), despite advances in supportive and critical care interventions.^(1)^

Mortality is often influenced by the severity of the underlying condition, the occurrence of multi-organ failure, and the timeliness of intervention. Key factors contributing to improved outcomes in recent years include better infection control, more effective conditioning regimens, enhanced immunosuppressive protocols, and improved pediatric intensive care unit (ICU) care. The use of invasive mechanical ventilation (IMV), inotropic support, or continuous renal replacement therapy (CRRT) has been identified as an independent risk factor for increased mortality in the pediatric ICU.^(1)^

While there have been advancements in ICU outcomes for pediatric haemato-oncology and HSCT patients in high-income countries over recent years, there remains a critical need to examine outcomes and trends in low- and middle-income countries (LMIC) settings.^(2)^ Historically, critically ill pediatric oncology and HSCT patients admitted to pediatric ICUs in LMICs face a very high risk, with a reported mortality rate of around 30.3%.^(3)^ A part of this important issue has been addressed in a Mexican study, in which capacity building to promote golden hour care has improved outcomes in febrile neutropenia.^(4)^

To improve outcomes, our center adopted a care improvement bundle in 2019 that included interventions to enhance communication, collaboration, and earlier recognition of clinical deterioration. This 10-year retrospective study compares outcomes between two cohorts: pre- and post-care improvement bundle implementation. The objective of this study was to evaluate the impact of a care improvement bundle focused on early intervention, communication, and multidisciplinary collaboration by comparing outcomes across two 5-year periods before and after its implementation.

## METHODS

**Figure 1:**
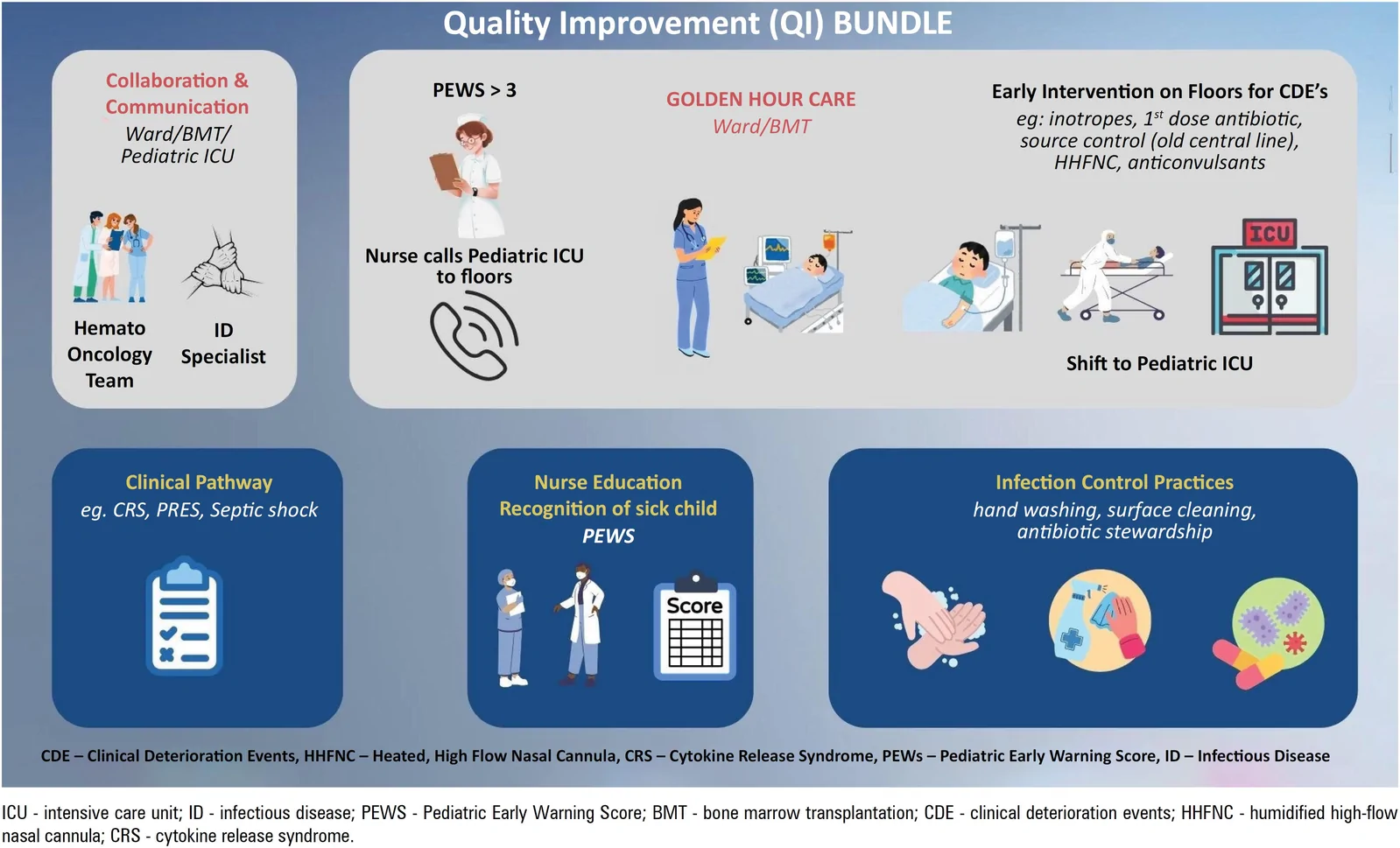
Care improvement bundle.

We are a tertiary care cancer and HSCT referral center in South India, serving patients with delayed diagnoses, failure to thrive, vaccine-related illnesses such as disseminated BCGosis in inborn errors of immunity, cardiac and liver iron overload in transfusion-dependent hemoglobinopathies, and those from financially challenged backgrounds. In 2018, we observed a mortality rate of up to 20% among pediatric hematology-oncology and HSCT patients in our onco-critical care pediatric ICU. Recognizing the urgent need for improvement, our pediatric oncology and pediatric ICU teams collaborated to develop a care improvement bundle, utilizing the golden hour principle and the Surviving Sepsis Campaign, emphasizing early recognition and intervention to improve outcomes.^(4,5)^ This bundle aimed to enhance multidisciplinary communication and collaboration for earlier detection and response to clinical deterioration events on the wards (Table 1).^(6)^ The focus was on five key areas:

– Collaborative care, introducing multidisciplinary co-rounds involving oncology, transplant, and other relevant subspecialties to formulate daily integrated care plans;
– Communication, encouraging early pediatric ICU consultation on the wards for patients at risk of life-threatening complications;
– Golden hour care, prioritising prompt initiation of resuscitation for clinical deterioration events (CDEs) while patients remain on the ward under pediatric ICU care, rather than waiting for an ICU bed;^(7,8)^
– Clinical pathways for common ICU diagnoses such as sepsis and cytokine release syndrome;
– Nurse education in recognising sick children using Pediatric Early Warning Score (PEWS) (Figure 1, Table 1)

**Table 1:** Care improvement bundle

Collaboration & communication	– Daily co-rounding of high-risk patients on floors by haemato-oncologists and the intensive care team to create integrated care plans	
	– Pediatric ICU team involvement in floors for patients at risk of serious events eg. posterior reversible encephalopathy syndrome, cytokine release syndrome	
	– Multidisciplinary discussions with infectious disease & other specialists	
	– Pediatric ICU team involved in pre-transplant stabilization of failure-to-thrive infants.	
	– Multidisciplinary family counselling	
PEWS	Nursing	
		– Use of PEWS
		– Recognition of shock-disproportionate tachycardia, low diastolic blood pressure (temperature, urine output, sensorium can be normal)
Golden hour care	Early initiation of resuscitation by the pediatric ICU team, including vasoactives for shock on the floors, rather than delaying interventions and losing precious time, until shifting to a pediatric ICU bed	
Clinical pathways	Algorithmic pediatric ICU protocols, personalized care	
	– Airway patency	
	– Breathing: heated, humidifier, high flow nasal cannula/ early intubation, if needed	
	– Circulation:	
		– Shock
		– Fluids: no rapid boluses, 5– 10mL/kg isotonic fluids over 1 hour. More only if losses are present or very negative
		– Early vasoactives (within the first hour of shock): noradrenaline (if low diastolic blood pressure), and adrenaline (if high risk /suspected or proven cardiac dysfunction/ malnourished). Initial vasoactives in the peripheral line. Target high normal blood pressures, if previously hypertensive
	– Steroids stress dose: hydrocortisone (if on steroids recently)	
	– Early source control: stop access to old central lines and removal	
	– Early dialysis to treat acidosis, prevent fluid overload: peritoneal dialysis in small children	
	Infection control:	
		– Antibiotics in the golden hour in septic shock (stocked in the ward)
		– Infectious disease consult for all patients on antibiotics
		– Surface cleaning, strict isolation practices, hand washing, perianal care
	Endotheliopathies:	
		– n-Acetylcysteine– with post-transplant cyclophosphamide (cardioprotective, antioxidant), for cystitis, mucositis, and sinusoidal obstructive syndrome
		– Hypertensive urgency/emergency – intravenous sodium nitroprusside, labetalol, prophylactic levetiracetam, hypertonic saline if drowsy
		– CRS
		– Early adrenaline. Anakinra/tocilizumab steroids (methylprednisolone) in engraftment CRS
		– Cardiac screening ECHO pretransplant, levosimendan for cardiac dysfunction
	Nutrition	
		– Semi-elemental diet in transplant patients if gut issues exist
		– Daily amino acid infusions for protein, if poor oral intake
		– Supplementation of phosphate, thiamine, trace elements, and magnesium
	Fluid balance	
		– Strict intake/output monitored every 6 hours
		– Avoidance of fluid overload. maintain euvolemia or slight negative
		– Daily complete blood count, blood gas (electrolytes, lactate)
	Early mobilization	
		– Passive/Active
		– On bed/out of bed
	Mother or family/nurse/physiotherapist	

PEWS - Pediatric Early Warning Score; ICU - inensive care unit; CRS - cytokine release syndrome; ECHO – Echocardiogram.

Figure created by the authors using the Canva Artificial Intelligence tool.

This care improvement bundle was implemented in 2019. Before implementing the bundle, oncology, HSCT, and critical care teams rounded separately and communicated mainly through progress notes. Oncology and HSCT providers usually did not consult the pediatric ICU team until patients became critically ill and required urgent transfer to the pediatric ICU. As a result, pediatric ICU interventions were delayed until the patient was physically transferred to the pediatric ICU in the pre-bundle period. After the care improvement bundle was introduced, the pediatric ICU team managed necessary resuscitation interventions once it was determined that the patient needed ICU care, regardless of the patient's location. Clinical pathways were only available for the post-bundle cohort. Before the bundle, nurses took vital signs and assessments on the ward every 6 hours and contacted physicians if they had concerns. In cohort 2, with PEWS integrated into the bundle, nurses were trained on a structured assessment tool, empowering them to contact physician teams in accordance with the PEWS algorithm.

We conducted a 10-year, single-centre, retrospective study from January 2014 to December 2023, using electronic health records to review charts of all patients aged 1 month to 18 years who required immediate pediatric ICU interventions in the wards and bone marrow transplant units before transfer to the pediatric ICU, as well as for direct admissions from the Emergency Department to the pediatric ICU. Patients were excluded if they were admitted to the pediatric ICU for elective procedures (e.g., extracorporeal photopheresis, red cell and plasma exchanges, critical site biopsies), postoperative care, end-of-life care, or if medical records were unavailable (84 patients in cohort 1 *versus* 72 in cohort 2). Consults seen by the pediatric ICU team that did not require intervention or pediatric ICU admission were also excluded.

Demographic and outcome data were collected by comparing pre- and post-care improvement bundle cohorts. Following the implementation of the care improvement bundle, we gathered information on CDEs occurring on the ward or in the pediatric ICU. These events were defined as tachycardia (disproportionate to temperature),^(9)^ respiratory distress,^(10)^ hypotension, hypertensive emergency, neurologic emergency, and severe bleeding.^(11)^ They were categorized based on the most severe presenting event, and the recorded events were mutually exclusive. These definitions were adapted from the paper by Bonafide et al.^(7)^ Mortality data before and after the care improvement bundle implementation (2014 - 2018 *versus* 2019 - 2023) were compared. Data on resource utilization, such as mechanical ventilation and renal replacement therapy (RRT), were also collected between the two cohorts.^(12)^ The number of pediatric ICU beds remained unchanged between the two cohorts.

### Statistical methods

Data were entered and analysed using SPSS Statistics version 21.0 (IBM Corp, Armonk, NY, USA). Descriptive statistics summarized baseline characteristics. Categorical variables were expressed as proportions and percentages. Comparative analyses for categorical variables were conducted using the chi-square test for independence. When cell counts were less than 5 in any category, Fisher's exact test was applied. The odds ratio (OR) with 95%CI was computed to assess the strength of the association between exposure variables and outcomes. Statistical significance was set at p < 0.05.

## RESULTS

In the pre-bundle implementation cohort (2014 - 2018), our hospital performed 516 HSCTs and cared for 336 new cancer patients. In the post-bundle implementation cohort (2019 - 2023), we performed 579 HSCTs and cared for 784 new oncology patients. Patient characteristics between the two cohorts were similar, except for a slight increase in infants undergoing transplants for metabolic and inborn errors of immunity (6.3% *versus* 7.3%), a doubling in the proportion of haploidentical transplants (20.3% *versus* 49.5%), and a significant rise in solid tumours, of which 21% were brain tumours in the later cohort (Table 2).

**Table 2:** Patient characteristics of two cohorts

		2014 - 2018	%	2019 - 2023	%
Transplant type					
	Matched family donor	270	52.3	191	33
	Autologous	30	5.8	33	5.7
	Haploidentical	105	20.3	287	49.6
	Unrelated donor	111	21.5	68	11.7
Underlying diagnosis					
	Hematological disorder	284	55	254	43.9
	Malignancy	122	23.6	157	27.1
	Inborn error of immunity	77	14.9	112	19.3
	Inborn error of metabolism	13	2.5	24	4.1
	Solid tumors	20	3.9	32	5.5
Sex					
	Male	343	66.5	358	61.8
	Female	173	33.5	221	38.2
Age group (years)					
	< 1	33	6.4	45	7.8
	1 - 5	216	41.9	216	37.3
	> 5	267	51.7	318	54.9
Total transplants		516		579	
Non-transplant - new oncology					
	Leukemia/lymphoma	214	63.7	320	40.8
	Brain tumor	9	2.7	90	11.5
	Other tumos*	113	33.6	374	47.7
Total		336		784	

* Neuroblastoma, Ewing's, Rhabdomyosarcoma, Wilms, Germ cell, Langerhans cell histiocytosis. Percentages are calculated from the total number of transplants (n = 516 for 2014 - 2018 and n = 579 for 2019 - 2023) and from the total non-transplant oncology cohort (n = 336 and 784, respectively). Age groups are defined as < 1 year, 1 to < 5 years, and > 5 years.

**Figure 2:**
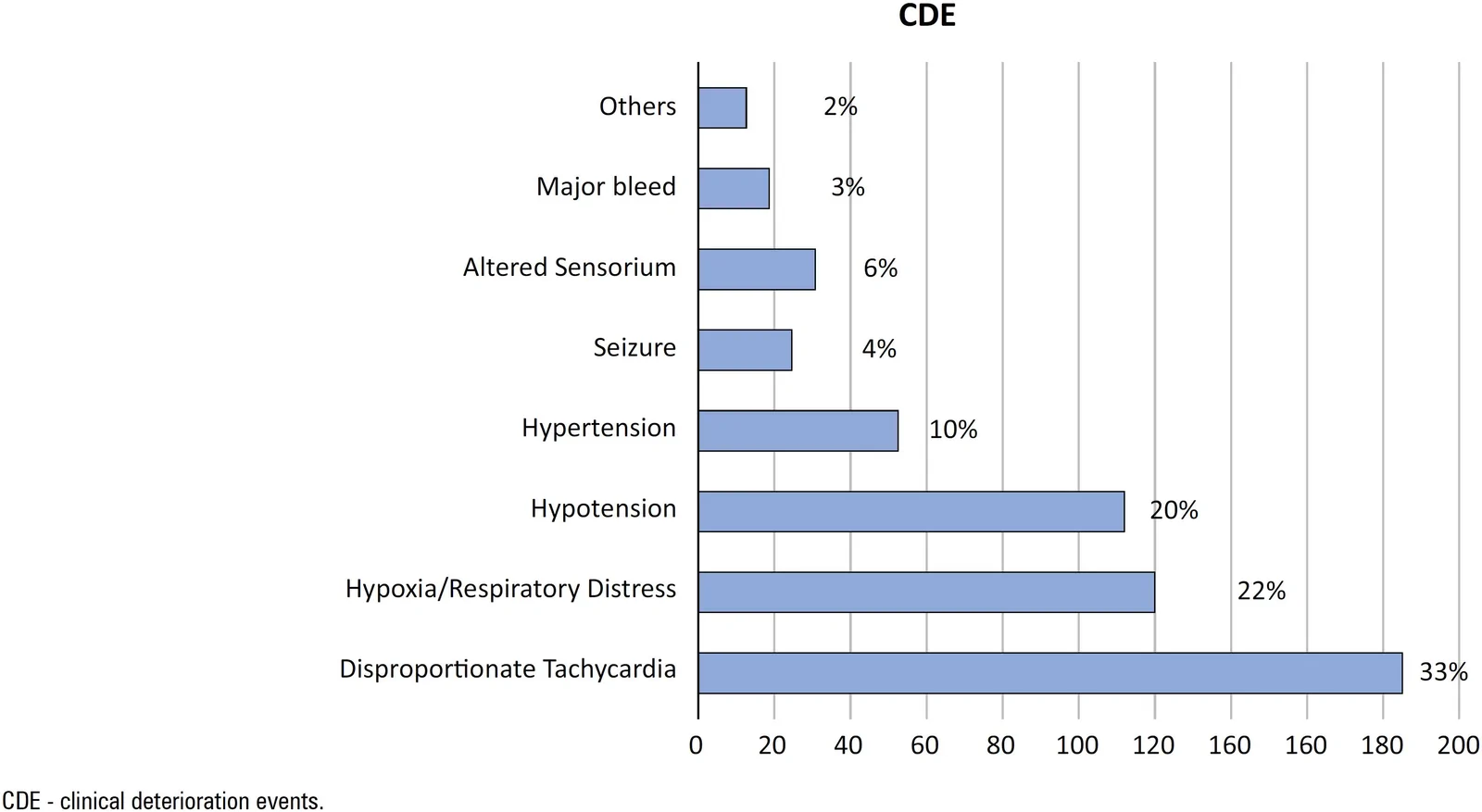
Distribution of clinical deterioration events on the inpatient floors.

In cohort 1, we had 533 pediatric ICU admissions, while in cohort 2, we had 760. We characterized the types of CDEs observed on the ward during cohort 2 after the bundle was implemented (Table 3). Although we cannot directly compare the cohorts due to unavailable CDE data for cohort 1, the most common CDEs in cohort 2 were disproportionate tachycardia, hypoxia/respiratory distress, and hypotension (Figure 2).

**Table 3:** Criteria for clinical deterioration events

CDE types	Operational definition	Number of events (n = 670)
Disproportionate tachycardia	HR > 95th percentile for age, unexplained by fever, pain, or dehydration	221 (33)
Hypoxia/respiratory distress	SpO_2_ < 92% on room air or new O_2_ requirement/non-invasive ventilation	147 (22)
Hypotension	SBP < 5th percentile for age or need for fluid bolus/vasoactive support	134 (20)
Hypertension	SBP > 95th percentile for age on two readings 6 hours apart	67 (10)
Altered sensorium	GCS < 13 or new-onset confusion/drowsiness	40 (6)
Seizures	Any witnessed or EEG-confirmed seizure requiring medical intervention	27 (4)
Major bleed	Clinically significant bleeding needing transfusion or hemostatic therapy	20 (3)
Others	Events not fitting above categories (e.g., arrhythmia, metabolic crisis)	14 (2)

For patients with multiple clinical deterioration events, only the most clinically significant event was considered for categorization (e.g., hypotension > tachycardia > hypertension). Percentages are rounded to the nearest whole number.

HR - heart rate; SpO^2^ - peripheral capillary oxygen saturation; SBP - systolic blood pressure; GCS - Glasgow Coma Scale; EEG - electroencephalogram.

For patients with multiple clinical deterioration events, only the most clinically significant event was used for categorization (e.g., hypotension > tachycardia, seizures > hypertension). Criteria for each clinical deterioration event and corresponding event counts are summarized in table 3.

Pediatric ICU and 100-day HSCT mortality outcomes significantly improved between the two eras. Pediatric ICU mortality decreased from 21.8% in cohort 1 to 11.4% in cohort 2 (p < 0.0001), with an absolute risk reduction (ARR) of 10.4% (p < 0.001) and a 47.7% relative risk reduction (RRR) (p < 0.001). There was also a significant reduction in the need for mechanical ventilation within 12 hours of pediatric ICU admission (12% *versus* 6.8%; p < 0.0001) and in mortality within the first 24 hours of pediatric ICU admission (6.4% *versus* 3.55%; p < 0.0001). Furthermore, the 100-day post-transplant mortality among all HSCT patients markedly decreased after implementing the care improvement (QI) bundle, dropping from 17.4% to 7.4% (p < 0.0001) (Table 4), despite the increased number of high-risk haplo-identical transplants in the later cohort.

**Table 4:** Outcome comparison of the two cohorts

Parameter	2014 - 2018 Pre-bundle cohort Pre PEWS	2019-2023 Post bundle cohort Post PEWS	p value
Total admissions in the PHO and BMT unit	4,000	5,007	
Total transplants	516	579	
Pediatric ICU admissions	533	760	< 0.11
Total mortality	116/4,000 (2.9)	87/5007 (1.73)	< 0.0001
Pediatric ICU mortality	116/533 (21.8)	87/760 (11.4)	< 0.0001
Mechanical ventilation	165/533 (30.9)	137/760 (18.0)	< 0.0001
Renal replacement therapy	79/533 (14.8)	33/760 (4.3)	< 0.0001
Ventilated < 12 hours of pediatric ICU admission	64/533 (12)	52/760 (6.8)	< 0.0014
Mortality < 24 hours of pediatric ICU admission	34/533 (6.4)	27/760 (3.55)	< 0.018
100-day transplant mortality	90/516 (17.4)	43/579 (7.4)	< 0.0001
Medical Emergency Team alarm rates	41/4,000 (1.02)	25/ 5,007 (0.50)	< 0.004

PEWS - Pediatric Early Warning Score; PHO - pediatric hematology oncology; BMT - bone marrow transplant; ICU - intensive care unit. Results expressed as n (number of patients) / N (total number of patients in the relevant denominator) and percentages.

Resource utilization decreased in cohort 2 (Table 4). In cohort 1, 30.9% of ICU admissions required mechanical ventilation, compared to only 18% in cohort 2. With an average daily cost of about $1,200 and an average mechanical ventilation duration of 3.29 days among 98 patients who did not require it, this could yield potential cost savings of $386,904. Similarly, the use of RRT decreased from 14.8% to 4.3% between the cohorts. In cohort 2, 79 patients avoided RRT at $600 per day for an average of 3 days, potentially saving $142,200 in our setting. These savings could be significantly higher in higher-income regions.

There was also a significant reduction in personnel utilization, along with a notable decrease in Medical Emergency Team (MET) alarm rates. These dropped from 1.04% to 0.50%, representing a 51.3% RRR, p < 0.004.

## DISCUSSION

Our 10-year study is among the largest cohorts of critically ill pediatric oncology patients in India. It demonstrates the possibility of achieving high survival rates for critically ill children in resource-limited settings through collaborative care and interdisciplinary communication, with PEWS for early detection of CDEs, prompt interventions during the golden hour on the floor, and protocol-based bundle management for specific complications.

Our findings reveal a significant decrease in pediatric ICU mortality, from 21.8% to 13.9% (p < 0.0001). This represents a substantial improvement, especially compared to previously reported mortality rates in LMICs, which range from 27% to 77%. Despite operating in an LMIC setting, the mortality rates at our facility are comparable to those in high-income countries, where rates range from 6.8% to 17.5%.^(13)^ This improvement in cohort 2 occurred despite a higher proportion of sicker children, including high-risk haploidentical and infant HSCTs performed for inborn errors of immunity and malignancy, as well as high-risk tumor cases such as brain tumors, neuroblastoma, and Ewing sarcoma, all associated with elevated mortality.^(13-16)^ Despite the high risk in HSCT patients in cohort 2, 100-day mortality improved for all HSCT patients, including those who did not require pediatric ICU admission, indicating that collaborative care can make a significant difference. There was a reduction in mortality within the first 12 hours of pediatric ICU transfer and a decreased need for invasive ventilation within the first 24 hours due to earlier pediatric ICU involvement. Among those who died, most deaths occurred among post-transplant patients (72% of all deaths) compared to non-transplant patients (28%). Post-transplant patients often require extended pediatric ICU stays due to transplant-related complications and infections. The collaborative care model has proven effective in reducing peri-transplant mortality, as reported by Reddy et al.^(6)^ In our cohort, the median pediatric ICU length of stay (LOS) for oncology and HSCT patients was 5.1 days ± 6.4 days, comparable to that of non-oncology patients. This finding is consistent with other multicentre studies from high-resource settings.^(17-24)^

Nurses’ implementation of PEWS in our study significantly reduced the need for MET activation and mortality in cohort 2. A recently published large meta-analysis and a multicentric Latin American study involving 32 pediatric oncology centers demonstrated a reduction in mortality from clinical deterioration events after implementing PEWS scoring.^(25,26)^ Implementing a structured protocol for recognizing and responding to early signs of clinical deterioration on the wards, along with immediate involvement of the pediatric ICU team, may prevent deterioration that often occurs during transfer delays. Our experience shows that empowering floor teams to initiate critical interventions- such as respiratory or circulatory support- and manage septic shock or hypertensive emergencies with pediatric ICU support while still on the floor can save valuable time. This approach reduces the need for invasive organ support and improves overall survival rates. It aligns with the broader principle of rapid response systems and could be especially impactful in resource-limited settings where pediatric ICU capacity is often stretched. By decentralizing critical care interventions to the floor level, golden hour care becomes a more proactive strategy, allowing timely, life-saving treatments even before a patient reaches the ICU.^(27,28)^

We believe that implementing multi-disciplinary co-rounding of all high-risk patients, even on the floors by oncology, HSCT, and pediatric ICU teams, has enabled us to develop a shared mental model of the patient's status more easily, leading to more realistic goals of care.^(29)^ Delayed treatments often result in more invasive procedures and higher costs. Our proactive care improvement bundle helped reduce the need for invasive interventions such as mechanical ventilation and RRT, resulting in cost savings. In resource-limited environments, establishing realistic expectations-especially during prolonged multiorgan dysfunction-is crucial not only for informed decision-making and ethical care but also to reduce unnecessary emotional and financial burdens on families, particularly in LMIC settings. Triage decisions should be individualized and based on clinical trajectory rather than diagnosis alone to ensure equitable access to life-sustaining therapies for this vulnerable but increasingly survivable population.

Although all components of the protocolized care improvement bundle were implemented, a formal objective scoring system to document compliance was not available, as this was a retrospective study. Another limitation is its single-center design. Changes in practice over time and shifts in the socio-economic demographics of our patients may also have influenced outcomes to some extent.

## CONCLUSION

This study highlights the importance of personalized, multidisciplinary care supported by simple, human-centered tools such as Pediatric Early Warning Score to identify golden hour signs. Golden hour care begins at the first signs of clinical deterioration, not after transfer to the pediatric intensive care unit. We implemented a straightforward, reproducible, scalable, and sustainable care-improvement bundle designed to Catch Them Early. The model strengthened communication and shared responsibility between the intensive care unit and oncology/haematopoietic stem cell transplantation teams, enabling earlier recognition and timely intervention for deterioration events occurring on the wards. In a resource-limited setting, this approach was associated with significant reductions in the need for high-cost, invasive therapies and improved survival.

## Data Availability

After publication the data will be available on demand to authors.
